# Exploring genetic sources of a decreased cognitive performance in psychometric schizotypy

**DOI:** 10.1192/j.eurpsy.2025.1531

**Published:** 2025-08-26

**Authors:** V. Plakunova, M. Alfimova, V. Golimbet

**Affiliations:** 1 Clinical Genetics Laboratory, Mental Health Research Center, Moscow, Russian Federation

## Abstract

**Introduction:**

Schizotypy is seen as subclinical part of the psychosis liability continuum. While several studies have attempted to confirm the relationship between schizotypal traits and genetic predisposition to schizophrenia using polygenic risk scores (SZ-PRS), the association of SZ-PRS with other features of schizotypal individuals resembling schizophrenia symptoms remains unexplored.

**Objectives:**

This study aimed to assess the contribution of SZ-PRS and PRS for other relevant traits to cognitive functioning in schizotypy.

**Methods:**

Healthy subjects (n=1468) were divided into low, negative, positive, and high mixed schizotypy groups based on a cluster analysis of the Schizotypal Personality Questionnaire data. Of them, 247 individuals had genome-wide information and completed a comprehensive cognitive battery, from which a cognitive index (CogI) was derived. PRS for schizophrenia, bipolar disorder, educational attainment, intelligence (IQ), neuroticism, and risk-taking were calculated with LDpred2-auto tool. The association of the CogI with PRSs was examined with stepwise multiple linear regression controlling for age and two ancestry-related principal components.

**Results:**

The groups differed in the CogI (p=0.015). The high schizotypy individuals (n=49) had a lower CogI than the low (n=73, p=0.01), negative (n=54, p=0.08), and positive (n=64, p=0.09) ones. SZ-PRS (β=-0.16, p=0.012) and IQ-PRS (β=0.13, p=0.014) predicted CogI in low schizotypy; IQ-PRS (β=0.42, p<0.001) in negative schizotypy; risk-taking PRS (β=-0.27, p<0.001) in positive schizotypy; and none of the PRSs predicted cognition in high schizotypy.

**Conclusions:**

We did not find traits whose PRS might explain the lower cognitive performance in high schizotypy. Thus, nongenetic factors deserve more attention in future research.

**Disclosure of Interest:**

None Declared

